# Integration of team-based learning and simulation-based learning in clinical education of infant feeding and swallowing assessment and management of speech-language pathology students: a retrospective pre-post intervention study

**DOI:** 10.1186/s12909-025-07721-7

**Published:** 2025-08-30

**Authors:** Connie Ching-Yin Kwan, Kelly Ann Weir, Raymond Fong, Valerie Jane Pereira, Thomas Law

**Affiliations:** 1 Department of Otorhinolaryngology, Head and Neck Surgery, The Chinese University of Hong Kong, Sha tin, New Territories, Hong Kong; 2 Institute of Human Communicative Research, The Chinese University of Hong Kong, Sha tin, New Territories, Hong Kong; 3 Audiology & Speech Pathology, Division of Medicine, Dentistry and Health Sciences, The University of Melbourne, Carlton, VIC Australia; 4 Allied Health Research, Royal Children’s Hospital, Parkville, VIC Australia

**Keywords:** Simulation training, Deglutition disorders, Speech-language pathology, Clinical, Education, Infant

## Abstract

**Background:**

Infant feeding and swallowing disorders are becoming more prevalent, resulting in an increased caseload for speech-language pathologists. However, clinical training for speech-language pathology students is often insufficient, primarily due to the vulnerability of the infant population and the absence of well-established training approaches. Simulation-based learning helps mitigate the challenges associated with frail clients, while team-based learning offers a systematic training framework. This study evaluates the effectiveness of combining these two approaches as a novel strategy for clinical education in this demanding clinical field.

**Methods:**

Using a single-group, pre-post intervention design, forty final year speech-language pathology master’s students participated in the study. They participated in an infant feeding and swallowing simulation clinic utilising the steps of team-based learning approach. Pre-and post- clinic knowledge test and level of clinical confidence were compared.

**Results:**

Students exhibited significant gains in knowledge following the integrated approaches clinic. Of the forty students, twenty-five students completed both the pre- and post-clinic survey and demonstrated a significant increase in their clinical confidence on managing infant feeding and swallowing disorders.

**Conclusions:**

The findings validate the positive impact of combining simulation-based learning with a team-based learning approach in improving students’ learning outcomes. This novel approach offers a better organised and hierarchical framework for teaching complex clinical areas in medical education.

**Clinical Trial Number:**

IRB/REC Reference No. 2024.392

**Supplementary Information:**

The online version contains supplementary material available at 10.1186/s12909-025-07721-7.

## Introduction

Infant feeding and swallowing disorders are prevalent, affecting 25% of premature infants [1], 47.2% of infants with neuromuscular disorders [2], and 85% of those with cerebral palsy [3]. Speech-language pathologists (SLPs) are essential members of swallowing management and early intervention teams for infants. In their everyday clinical practice, they often manage cases of infants with feeding and swallowing challenges. In SLP clinical education, it is vital to provide training for the assessment and management of infant feeding and swallowing difficulties. Nevertheless, there is a frequent absence of training in this domain [4] due to this practice area usually being managed by experienced, specialised SLP in health settings, and the challenges associated with identifying clients for student placements [5, 6].

### Simulation-based learning

Simulation-based learning (SBL) is becoming popular in medical education. This educational method simulates real-world situations in a controlled setting, enabling students to develop their skills without endangering patients [7]. According to ‘Implementing the Standards of Best Practice for Simulation’ by Rutherford-Hemming, Lioce [8], effective SBL extends beyond the simulation event; it constitutes a structured pedagogical process involving three critical phases: pre-briefing, simulation scenario, and debriefing. The procedures of SBL are described in Table 1. SBL has been proven to significantly improve students’ clinical skills by facilitating intentional practice, practical learning, and the acquisition of intricate diagnostic and treatment abilities [9]. It ensures an identical and assured exposure for students [10]. This may be especially advantageous for novice clinicians.

**Table 1:** Procedures of simulation-based learning

Step	Component of Simulation-based learning	Description
1	Pre-briefing	This is essential for defining the simulation’s aim and ensuring a psychologically secure setting in which students feel at ease participating and making errors. In this phase, facilitators evaluate learning objectives, elucidate relevant terminology, and establish standards for professional integrity and engagement.
2	Simulation scenario	This serves as the practical element in which students actively participate in the clinical case.
3	Debriefing	This is the most crucial phase for acquiring knowledge. It is a systematic, facilitator-guided reflective process in which students evaluate their activities, examine their clinical reasoning, obtain constructive feedback, and integrate their learning to connect simulation with real-world practice.

In the domain of speech-language pathology clinical education, use of SBL has been investigated in stuttering management, augmentative and alternative communication, and paediatric dysphagia [11–13]. This method has demonstrated efficacy in enhancing student confidence, alleviating anxiety, and improving readiness for clinical placements [14, 15]. Ward, Hill [13] reported that the integration of human patient simulation in the education of paediatric feeding problems markedly improved the knowledge, abilities, and confidence of SLP students.

SBL has been demonstrated to enhance students’ reflective abilities. Debriefing following high-fidelity simulation improves the reflective thinking capacity of nursing students; however, facilitators have to focus on establishing a conducive atmosphere to foster this reflective thinking [16]. When accompanied by suitable scaffolding, SBL may enhance the development of intricate skills, including reflective practices, within higher education sectors [9]. This reflective capacity is essential for understanding infant dysphagia, considering students’ limited exposure to the topic and the need for self-directed study post-graduation.

### Team-based learning

Team-based learning (TBL) offers an active, organised method of small group learning applicable to large courses. The implementation comprises distinct phases as outlined in Table 2: pre-class preparation, readiness assurance testing, problem-solving activities, and instant feedback [17]. A rising number of healthcare faculties worldwide have implemented TBL in various combinations across different environments and subject areas [18, 19]. It enhances exam performance, communication, and motivation among medical students [20]. Furthermore, it consistently enhances academic outcomes by transitioning the instructional emphasis from knowledge transmission to knowledge application, while also addressing various professional competencies that cannot be attained or assessed through lecture-based instruction in medical education [21].

**Table 2:** Steps of team-based learning

Step	Component of Team-based learning	Description
1	Pre-class preparation	View pre-class micromodule in the format of video
2	Individual readiness assurance test (IRAT)	Complete multiple choice questions regarding the micromodule individually before class
3	Team readiness assurance test (TRAT)	Taken in class using identical questions as the IRAT with their team members in class
4	Immediate feedback or clarification	Instant feedback on team response based on results of IRAT & TRAT
5	Clinical problem-solving activities	Practice decision-making and judgements through case study in group format
6	Closing	Reflect own learning with facilitator, clarify misunderstanding and summarizing take-home messages

### Integration of team-based learning and simulation-based learning

The clinical management of infant dysphagia is a high-stakes practice area where accurate evaluation is critical. This patient population is often medically fragile, and mismanagement can lead to fatal health consequences, including aspiration pneumonia—a condition associated with significant mortality [22]. The profound vulnerability of these infants, combined with the potential for such adverse outcomes, creates a stressful clinical environment that can be a source of significant anxiety for novice clinicians [5]. To address this, SBL supports students to be more equipped to manage actual clinical scenarios upon entry to practice [13]; while learning in a team-based approach may reduce anxiety and encourage students’ engagement in the clinical scenario [17].

Despite the advantages of simulation, Bae et al. [23] identified three primary factors hindering learning at different stages of simulation. Challenges arose in integrating theoretical information during the prebriefing stage, restrictions in clinical reasoning due to psychological stress during the scenario phase, and constraints in reflective thinking during the debriefing phase. The characteristics of TBL, encompassing collaborative learning, prompt feedback, and peer evaluation via discussion [17], may effectively address the shortcomings of SBL.

Although SBL has been proven to enhance clinical competences across several areas of speech-language pathology, and TBL has shown effectiveness in fostering collaborative learning and critical thinking, there is little research on the effective integration of both approaches. Current research has predominantly concentrated on individual approaches, neglecting to investigate the possible benefits of their integration. This study aims to address this deficiency by examining the effects of an integrated SBL and TBL approach as a clinical teaching pedagogy for SLP students in the area of the assessment and management of infant dysphagia. The results of this study potentially offers important insights into the enhancement of clinical education of SLP students.

### Study objectives

The present study aims to:

1. Determine the change in SLP students’ confidence before and after undergoing the combined SBL and TBL approach in infant feeding and swallowing assessment and management.
2. Evaluate the change in knowledge among SLP students following the combined learning approach.

### Hypothesis

1. SLP students will report higher levels of confidence in performing infant dysphagia assessments and management tasks before and after undergoing the combined SBL and TBL approach in infant feeding and swallowing clinic.
2. Students will demonstrate a significant gain in knowledge and application related to infant feeding and swallowing disorders after participating in the combined SBL and TBL approach.

## Method

This study adopted a retrospective single-group, pre-post intervention design. The decision to conduct the research was made after the course concluded, and students were therefore not explicitly informed at the time of data collection that their responses might be used for future research. The knowledge tests were mandatory course activities, while the confidence survey was voluntary and presented as a tool for instructor feedback. Both the surveys and tests were part of a standard, low-stakes educational program intended for formative feedback for the instructor, not for summative grading for the students.

Quantitative survey data before and after the placement and test results during the micromodule and after the placement were obtained from Qualtrics (Qualtrics, Provo, UT) and Grade centre of the university online learning system ‘Blackboard’ (https://blackboard.cuhk.edu.hk) respectively. All data was collected in a ‘de-identified’ manner, with no access to personally identifiable information of the students. Therefore, the study was granted with waiving of consent form. This study was conducted in accordance with the Declaration of Helsinki and received ethical approval from the Joint CUHK-NTEC Clinical Research Ethics Committee (approval number: [2024.392]).

### Participants

The study was planned to retrospectively identify data of all students from the final year of a 2-year Master of Science in Speech-Language Pathology in the Chinese University of Hong Kong. Convenience sampling was adopted; therefore, the number of students in the course during the study period determined the sample size. Students who had registered for the course SLPA5305 Work Integrated Learning & Special Clinic II and completed the 4-hour Paediatric Feeding Clinic between 1 st April, 2024, and 31 st July, 2024 were included. Exclusion criteria included students who dropped the course, and students with incomplete test records. All students were adults and no age range limit or gender selection was included.

All students were native Cantonese speakers studying in English. The study involved two distinct data retrieval components. The first was the knowledge assessment (IRAT/TRAT), a mandatory part of the course curriculum, for which data were collected from the entire cohort (*n* = 40). The second was a voluntary pre- and post-course survey on confidence. A self-selected subgroup of 25 students (62.5%) completed both surveys; this subgroup had an age range of 23 to 40 years (M = 27 years, Median = 26 years) and consisted of 2 male (8%) and 23 female (92%) students. Consequently, the analysis of knowledge gain is based on all 40 participants, while the analysis of confidence is based on the subgroup of 25.

### Procedure

**Fig. 1:**
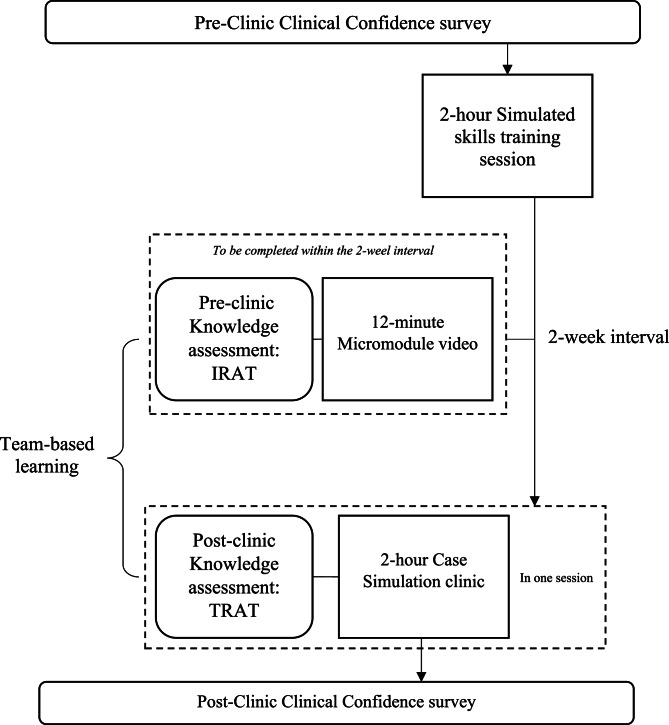
Flow of paediatric feeding clinic and outcome measures. IRAT: Individual readiness assurance testing, TRAT: Team readiness assurance testing

The paediatric feeding clinic consisted of three components: a 2-hour simulated skills training session, a 12-minute micromodule video with IRAT, and a 2-hour case simulation with TRAT using TBL approach (see Fig. 1). This multi-component structure represents an adapted application of the TBL and SBL frameworks, designed to fit within existing curriculum constraints. There was a 2-week interval between the simulated skills training sessions and case simulation clinic, so as to allow adequate time for students to view the micromodule videos and prepare for the clinic.

#### Simulated skills training session

This 2-hour simulated skills training session covered the basic hands-on skills for infant feeding and swallowing disorders. Details of the session were shown in Table 3. Students were assigned in a group of 9–10 students to 1 instructor. Eight components were included in the session, ranging from general infant caring skills to infant dysphagia assessment and management skills. This session prepared students for the simulation clinic and served as a ‘clinical bridging’ to bridge theories taught in the lecture to practice.

**Table 3:** Details of simulated skills training session

Area	Details
Duration	2 h
Instructor-to-students ratio	1:9–10
Format	Simulation of different infant feeding tasks
Materials	Infant mannequins, milk bottles and teats of different brands, swaddling clothes
Content (Skills covered)	General infant caring skills 1. Handling skills (holding infant) 2. Milk bottle assembling​ 3. Swaddling
	Infant dysphagia assessment skills 4. Non-nutritive sucking assessment
	Infant dysphagia feeding and management skills 5. Bottle feeding skills 6. Burping 7. Elevated side-lying 8. Pacing

#### Micromodule

After attending the simulated skills training session, students would receive a 12-minute micromodule video, in which they learn the observation skills during bottle feeding for two infants. Students were required to view the micromodule individually online and completed a knowledge test after viewing the micromodule. Infant dysphagia assessment skills, including counting suck: swallow ratio, sucking rate, number of sucks per sucking burst and signs suggestive of respiratory compromise were demonstrated in the video. A knowledge assessment test, Individual Readiness Assurance Test (IRAT) [17], was adopted after viewing the micromodule video, on the university online learning platform ‘Blackboard’. This part prepared students for the case simulation clinic 2 weeks later.

#### Case simulation clinic with team-based learning approach

After a 2-week interval, students attended a 2-hour case simulation clinic using TBL approach in a group of 5–6 students. Before the start of the simulation, the knowledge assessment test in group format, Team Readiness Assurance Test (TRAT) [17] using the same questions as in the IRAT, was implemented. Immediate feedback and clarification were given based on the results of IRAT and TRAT. This aimed to consolidate the knowledge learnt and clarify misconceptions before the simulation started.

The clinic format was a hybrid simulation mode, which involved the use of one low fidelity infant mannequin and one simulated caregiver. It was conducted in a real clinic room at the University speech therapy clinic to improve immersion and contextual learning. During the simulation clinic, students played the role of SLP and were required to implement a clinical bedside swallowing evaluation for an infant aged 35 days and complaint of choking. The specific learning goals include: (a) conduct a comprehensive case history interview with the simulated caregiver; (b) perform an appropriate oromotor and non-nutritive suck assessment; (c) accurately observe and interpret the infant’s physiological and behavioural cues (e.g., desaturation, coughing) during a feeding trial; and (d) collaboratively select and trial at least one appropriate intervention strategy (e.g., pacing, elevated side-lying).

Dynamic technology integration was employed. Computerized application of paediatric patient monitor simulator, namely PEDS VITALS [24], was used to provide the instant display of vital signs of saturation level, heart rate and respiratory rate of the low fidelity mannequin throughout the session. The monitor simulator was controlled with another tablet device by the instructor. Additionally, the clinical signs of the infant mannequin were narrated by the instructor verbally to the students (e.g. “The infant is coughing, Pulse oximetry is reading 80% SpO_2_.”).

A notable feature of this simulation clinic was the incorporation of a team-based learning method that fostered discussion and collaborative learning. A transition towards collaborative practice is occurring. Rather than traditional individual clinician simulations, the clinic operated on a team-based model, emphasising collaborative clinical reasoning and shared decision-making as the primary learning objective.

**Fig. 2:**
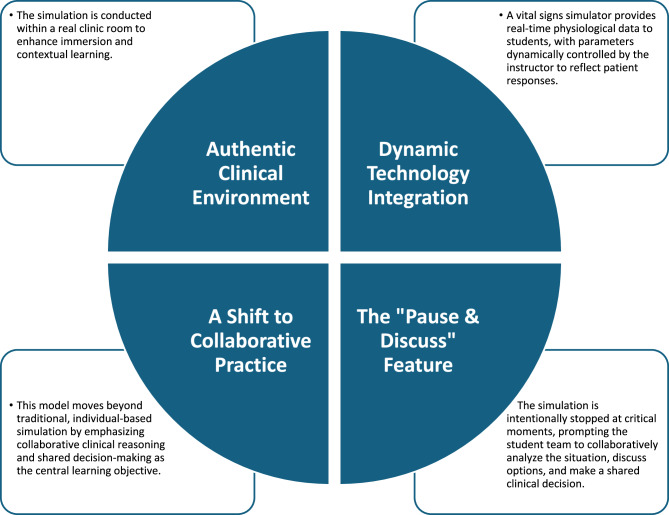
Key features of case simulation clinic

During the simulation (i.e. step 5: Clinical problem-solving activities in TBL), the “Pause & Discuss” feature was utilised. Students were permitted to ‘stop’ the simulation at their discretion to confer with their peers regarding the subsequent steps of assessment or intervention. They were encouraged to use a ‘think aloud’ protocol to verbalize their clinical reasoning as a team. Feedback during this step was primarily peer-driven, facilitated by the unique ‘pause and discuss’ feature, with formal, summative feedback being reserved for the debriefing phase (Step 6). For example, when students noted desaturation while feeding using a large-orifice teat, they may ‘stop’ the simulation to deliberate on further interventions before doing another experiment. Figure 2 illustrates the key features of the case simulation clinic.

At the end of the session, debriefing was held. Students could reflect on their own clinical skills, clarify any misunderstandings and summarize the case study. For the detailed steps of TBL and SBL in paediatric feeding simulation clinic, refer to Table 4.

**Table 4:** Detailed steps of team-based learning and simulation-based learning in paediatric feeding simulation clinic

Step	Component of Team-based learning	Component of Simulation-based learning	Description
1	Pre-class preparation	Not applicable	View pre-recorded micromodule on infant dysphagia assessment skills before the clinic
2	Individual readiness assurance test (IRAT)	Not applicable	Complete 6 multiple choice questions regarding the micromodule individually before class
3	Team readiness assurance test (TRAT)	Not applicable	Complete the same 6 multiple choice questions as in the IRAT with their team members after discussion
4	Immediate feedback or clarification	Pre-briefing	Instant feedback on team response based on results of TRAT by the instructor. This is followed by the pre-briefing for the simulation, which establishes a safe learning environment, outlines objectives, and familiarise students to the scenario and mannequin.
5	Clinical problem-solving activities	Simulation scenario Adopt a 2- hour hybrid simulation model utilizing one low-fidelity infant mannequin and one simulated caregiver	Practice decision-making and judgements through a simulated case of 35-day-old infant complaint of choking in a group format. Allow students to collaborate as a team of speech-language pathologists while having the autonomy to ‘pause’ the simulation for peer discussions by ‘Talk aloud’ protocol regarding subsequent assessment or intervention steps Learning Goals: - Conduct case history interview - Perform oromotor & non-nutritive suck assessment - Observe & interpret infant’s cues during feeding - Select & trial appropriate interventions
6	Closing	Debriefing	A structured, facilitator-led debriefing where students verbally reflect on their clinical skills, analyze decision-making processes, clarify misunderstandings, and summarize key take-home messages to consolidate learning.

### Data collection & measures

Data was extracted via two systems to assess students’ knowledge and clinical confidence on infant dysphagia. All data was collected in a ‘de-identified’ way, ensuring that no personally identifiable information about the students is accessed or disclosed. To ensure confidentiality and enable pre-post linking, a non-identifiable code was assigned to each student for data matching, after which all data were fully de-identified by an administrative staff member not involved in the research prior to analysis. Students’ demographic data of age and gender were provided by the division clerical staff in an anonymous format.

#### Knowledge assessment (IRAT and TRAT)

Students’ knowledge was assessed using the assessment method of TBL assessment. The test consisted of 6 multiple-choice questions regarding infant dysphagia assessment (See Appendix A). The IRAT and TRAT questions were identical, following the framework of TBL by Burgess, van Diggele [17], in order to precisely assess students’ knowledge change. Students were required to complete the IRAT after the simulated skills training session and viewing the micromodule video; and complete the TRAT at the end of the simulation clinic (See Fig. 1). Scores of IRAT and TRAT were extracted from the university online learning system ‘Blackboard’ (https://blackboard.cuhk.edu.hk).

##### Clinical confidence survey

Students’ clinical confidence was measured via a self-rating survey. Students were asked to fill in the survey before the simulated skills training session and two weeks after the clinic to measure their clinical confidence. Each student was given an unidentified identity number for the course to ensure anonymity. The survey utilized the Qualtrics (Qualtrics, Provo, UT) platform and consisted of 20 questions with a 5-point Likert scale response (See Appendix B). The questions were developed based on five out of seven areas in Competency Based Occupational Standards (CBOS) as students’ clinical competency was assessed by CBOS throughout the clinical placement curriculum. Each area comprised of 4 questions regarding students’ confidence on that area. Students rated on a 1–5 scale (1 = not confident at all to 5 = very confident). All survey responses were anonymous. Only data of the eligible students was collected through Qualtrics.

### Data analysis

Descriptive statistics were used to summarize the demographic data of the students. As the data were retrospective, their normality of variation was unknown. Therefore, the normality of variations between pre- and post-knowledge test scores were assessed using the Kolmogorov-Smirnov test, to determine whether to use a parametric or non-parametric test. As calculated, the difference between pre- and post-clinic knowledge test scores, D(40) = 0.21, *p* <.001, was significantly not normally distributed. Therefore, non-parametric related sample Wilcoxon signed rank test was adopted. Due to small sample size of subgroup (*n* = 25) in both knowledge test scores and level of clinical confidence, non-parametric related sample Wilcoxon signed rank test was employed for its analysis using IBM SPSS Statistics (Version 28). Statistical significance was set at a *p*-value of < 0.05 for the score of knowledge test and total score of clinical confidence questionnaire. Bonferroni correction was adopted to adjust the significance level for the five sub-categories under the clinical confidence questionnaire, and their corresponding significance level was therefore set as 0.01 (0.05/5) to reduce type I error. Effect size, r, was calculated and compared using Cohen’s d classification to measure the magnitude of the difference before and after the clinic [25].

## Results

### Participants

The study extracted data of all students (*n* = 41) from the final year of a 2-year Master of Science in Speech-Language Pathology who completed the 4-hour Paediatric Feeding Clinic between 1 st April, 2024, and 31 st July, 2024. Exclusion criteria were students who dropped the course (*n* = 1), and students with incomplete test records (*n* = 0), constituting a sample size was 40. The students’ age range was 24 to 40 years (M = 27 years, Median = 27 years), with 3 male (7.5%) and 37 female (92.5%) students. All students had attended a 3-hour lecture on paediatric dysphagia in the preceding academic year during the academic course: Dysphagia, but all of them had no prior clinical experience of infant feeding after the 3-hour lecture.

### Change in knowledge

To clarify the sequence of events, the following knowledge outcomes were measured after the completion of the TBL module but prior to the students’ engagement in the SBL component. Considering the results for the whole class (*n* = 40), students’ knowledge on observation skills during bottle feeding assessment after the clinic (Mean = 5.9) was significantly higher than that before the clinic (Mean = 3.5), z = 5.23, *p* <.05, *r* =.83. After excluding the students without filling in the post-clinic questionnaire, similarly, their knowledge test score (Mean = 6) was significantly higher than that before the clinic (Mean = 3.3), z = 4.24, *p* <.05, *r* =.85 (See Table 5).

**Table 5:** Knowledge test result of IRAT and TRAT scores

	Pre-clinic (IRAT)		Post-clinic (TRAT)		Test statistic, Z	Sig.^*^	Effect size
	Mean	SD	Mean	SD			
Whole class (*n* = 40)	3.5	1.26	5.9	0.30	5.23	< 0.001	0.83
Subgroup^1^ (*n* = 25)	3.3	1.25	6	0.00	4.24	< 0.001	0.85

^1^Data from the subgroup (*n* = 25) of the total cohort (*n* = 40) who voluntarily completed the pre- and post-clinic confidence survey

* The significance level is 0.05

### Change in clinical confidence

Regarding students’ clinical confidence level before and after the clinic, students’ overall clinical confidence after the clinic (Mean = 62.1) was significantly higher than that before the clinic (Mean = 30), z = 4.37, *p* <.05, *r* =.87. Details of the scores in different clinical competency areas were presented in Table 6.

**Table 6:** Student confidence levels (*n* = 25)

	Pre-clinic		Post-clinic		Test statistic, Z	Sig.	Effect size
	Mean	SD	Mean	SD			
Total score	30	10.89	62.1	13.56	4.374	< 0.001^*^	0.8748
Assessment	6.3	2.35	12.8	2.80	4.342	< 0.001^**^	0.8684
Analysis and interpretation	5.5	2.20	12.6	2.74	4.296	< 0.001^**^	0.8592
Planning evidence-based practices for Pediatric Dysphagia	5.8	2.44	12.4	3.00	4.387	< 0.001^**^	0.8774
Implementation of Pediatric Dysphagia Practice	6.1	2.44	12.2	2.89	4.210	< 0.001^**^	0.8420
Managing Pediatric Dysphagia Services	6.3	2.70	12.0	3.10	4.212	< 0.001^**^	0.8424

*p*-values reflect a Bonferroni correction for multiple comparisons

*p <.05

**p <.01

## Discussion

The aim of this study was to describe the implementation of an integrated pedagogical module using TBL and SBL, and to evaluate its effect on the knowledge and clinical confidence of final-year SLP students. Its primary contribution is the creation of an innovative hybrid pedagogical paradigm and an initial demonstration of its effectiveness for enhancing student knowledge and confidence in infant dysphagia care, a field that has been historically overlooked in clinical education. We hypothesized that SLP students would report higher levels of confidence and have a significant increase knowledge score after undergoing the combined SBL and TBL approach in infant dysphagia training. Referring to the results, our hypotheses were confirmed with a large effect size. Students rated themselves with higher clinical confidence level in all clinical competency areas.

### Change in level of knowledge

A key contextual factor for this study is that the students’ only prior formal learning on this topic was a single three-hour lecture that occurred in preceding academic year, over a year before the intervention. In the interim, students had no clinical exposure to infant feeding. At the start of the clinic, their baseline knowledge was assessed via the IRAT. Following the TBL/SBL module, the TRAT was administered. The findings indicated a statistically significant enhancement from IRAT to TRAT ratings. This demonstrates the efficacy of the skills lab training, the pre-class self-study micromodule video, and the particular aspect of discussion in step 3 of TBL (discussing with teammates during TRAT) in facilitating students’ acquisition of knowledge on clinical observation in infant swallowing and feeding assessment, which is crucial to building a knowledge foundation for students’ clinical practice.

The substantial rise in knowledge scores from the IRAT to the TRAT aligns with extensive research supporting the effectiveness of active learning strategies in health professions education. The application of TBL has demonstrated enhanced knowledge results relative to conventional lectures [19]. Likewise, the enhancement in post-clinic knowledge scores also indicates the efficacy of the pre-class video (which is step 1 of TBL). This corresponds with the findings of Ferguson and Estis [26], indicating that videos facilitated students in the efficient application of infant feeding assessment to cases and the enhancement of critical thinking abilities prior to engaging in relevant clinical practicum. After all, simulation offers a framework for the application of knowledge inside a genuine clinical setting [27]. Our findings align with Broadfoot and Estis [28] work, which proved a possible way for training entry-level clinical students to assess feeding skills in infants.

### Clinical confidence

Clinical confidence might be defined as the psychological state characterised by assurance or certainty in a clinical domain. This study demonstrated a notable enhancement in students’ clinical confidence about several aspects of paediatric feeding and swallowing under the Competency Based Occupational Standards (CBOS), which constitute the educational prerequisites for professional membership eligibility in Hong Kong [29]. A minimum two-fold rise was seen in the raw score for confidence in assessment and management techniques, as well as in the formulation of rationales for various assessment tasks and intervention strategies. Furthermore, the results demonstrated a large effect size, with Cohen’s d exceeding 0.8. This substantial rise aligns with prior research on SBL on the enhancement of students’ clinical confidence in different clinical areas [12–14].

This increase in clinical confidence may be attributed to two possible explanations. According to Gottlieb, Chan [30], confidence is contingent upon certain tasks and is influenced by contextual factors and personal experiences. The simulation clinic was developed by experienced speech-language pathologists specializing in paediatric feeding and swallowing to address the clinical needs of assessing and intervening paediatric feeding disorder. The authenticity of the clinical situation in simulation, along with the specific observational skills acquired from micromodule viewing (step 1 of TBL), enhances students’ confidence in this area. Moreover, confidence should be considered with knowledge [30]. The notable enhancement in knowledge following TBL (pre-clinic mean score: 3.5; post-clinic mean score: 5.9) augmented the efficacy of bolstering students’ clinical confidence.

### Progressive learning for knowledge application

A key strength of this pedagogical approach is its clear, step-by-step design, which systematically moves students from learning foundational concepts with the micromodule to applying them directly in a simulated clinical case. This structured progression led students from foundational knowledge acquisition to practical clinical application and ultimately to reflective consolidation.

Our method commenced with the establishment of a knowledge base utilising core TBL principles (Steps 1–3). Students participated in pre-class preparation before being evaluated through the IRAT. This is then followed by collaborative learning within the TRAT, a sequence recognised as a feature of TBL for facilitating structured learning [17]. The theories and knowledge acquired in these steps seem to tackle one of the issues of SBL identified by Bae—specifically, the difficulties in integrating theoretical knowledge during the pre-briefing phase [23].

The next step then shifted from knowledge acquisition to application at a critical bridging phase (Step 4). The instructor-led clarification on TRAT items reinforced knowledge, while the pre-briefing set expectations for students prior to the simulation scenario [31]. This established the foundation for the clinical problem-solving exercises (Step 5), during which students engaged in a high-fidelity clinical scenario. A fundamental instructional element was the “pause and think aloud” approach, which enabled students to participate in real-time, collaborative clinical reasoning. This proactive problem-solving transcends simple practice and cultivates the metacognitive skills essential for proficient clinical judgement [32]. This may also address another impediment to successful simulation identified by Bae, namely that students may experience difficulties in clinical reasoning due to psychological stress during the scenario phase [23].

The learning sequence culminated in a structured, facilitator-led debriefing (Step 6) conducted by an experienced SLP. This reflective approach adheres to the ‘Healthcare Simulation Standards of Best approach of The Debriefing Process’ [33], as it involves the analysis of clinical experience, facilitating enduring learning and enhanced future performance. The deliberate, multi-faceted advancement—from knowledge to application to reflection—explains the notable enhancements observed.

### Limitations and future directions

Although the study yields encouraging findings, specific limitations must be acknowledged. This study is conducted at a single site with a relatively small sample size. The sample was obtained from a single cohort of students at a single university, and our analysis does not account for student attrition, which is another limitation. This limits the representativeness of our findings, as these students may possess unique academic backgrounds or characteristics not representative of the wider SLP student population. Therefore, caution should be exercised when generalizing these findings to students in different institutions or contexts.

Additionally, a primary limitation of this study is its single-group, pre-test/post-test design, which lacks a control group. The absence of a control group precludes us from conclusively ascribing the observed improvements in knowledge and confidence only to our hybrid intervention. Other factors, such as concurrent coursework or the Hawthorne effect—where participants’ awareness of being observed affects their behavior—may have contributed to the positive outcome [34]. Future research utilising a randomised controlled trial is essential to delineate the distinct benefits of our TBL-SBL paradigm in contrast to conventional teaching approaches.

Furthermore, our study implemented a modified integration of the TBL and SBL frameworks instead of a ‘pure’ integration. This adaptation, while pragmatic, is a limitation. However, from a continuous quality improvement (CQI) perspective, these modifications provide explicit guidance for future research aimed at refining and optimising the model. Future iterations should investigate the fidelity to TBL principles. A significant limitation was the pre-existing 3-hour lecture, which resulted in students not obtaining knowledge only from the pre-class micromodule. Future research should investigate the necessity of this lecture to determine the true impact of the TBL preparatory materials, especially on areas involving hands-on skills, such as swaddling skills in this study. Also, the current study focused on immediate changes in knowledge and confidence rather than higher-order clinical competence. Future study ought to incorporate direct assessments of clinical competencies and utilise longitudinal methodologies to monitor the enduring effects on graduates’ performance and confidence in practical clinical settings.

A methodological concern involves several problems with measurement. Our use of the individual IRAT score as the pre-test and the team-based TRAT score as the post-test is one example. While fundamental to the TBL process, this is a limitation because it precludes isolating individual knowledge gain. More broadly, our study relied on limited outcome measures, focusing on low-level knowledge rather than clinical competence, and lacked measurement at critical time points, such as an individual post-test after the simulation. Future research may include a conclusive, individual post-test following the debriefing to better analyse the impacts of the different components of this integrated pedagogical approach. Moreover, future research should examine the long-term impacts of this integrated strategy on SLP students’ clinical competence, confidence, and knowledge change across time. Longitudinal studies assessing graduates’ performance in clinical environments may provide valuable information.

Despite the aforementioned limitations, the pedagogical framework presented in this study holds considerable potential for generalizability. Although the content specifically addressed infant dysphagia, the basic concepts of the hybrid TBL-SBL model—especially the use of collaborative ‘Pause & Discuss’ intervals during practical simulations—are readily applicable to other contexts. This model offers a pragmatic framework for educators in various clinical fields (e.g., nursing, medicine, physiotherapy) aiming to cultivate collaboration and collaborative decision-making abilities. The research provides fresh insights by illustrating that this hybrid approach is both viable and successful in cultivating the knowledge and confidence necessary for intricate clinical situations. Subsequent research should endeavour to replicate this model in other student demographics and across several therapeutic subjects to enhance its validation and adaptability.

## Conclusion

In conclusion, this study successfully demonstrated that a novel, integrated TBL and SBL module was associated with significant improvements in SLP students’ knowledge and self-reported confidence in managing paediatric dysphagia. The results indicate that the consolidation of knowledge acquisition by TBL and clinical skill application through SBL offers a systematic approach to augment students’ knowledge and confidence. This pedagogical approach offers a promising model for bridging the critical gap between theoretical knowledge and its practical application, thereby better preparing emerging clinicians for the demands of real-world clinical practice.

## Supplementary Information

Supplementary Material 1.

Supplementary Material 2.

## Data Availability

The author confirms that all data generated or analysed during this study are included in this published article.

## Abbreviations

SLPs: Speech-language pathologists
SBL: Simulation-Based Learning
TBL: Team-based learning
