# Supplementary material for: Integration of team-based learning and simulation-based learning in clinical education of infant feeding and swallowing assessment and management of speech-language pathology students: a retrospective pre-post intervention study
Source: BMC Med Educ. 2025 Aug 30;25:1228. doi: 10.1186/s12909-025-07721-7 (PMC12398139; doi:10.1186/s12909-025-07721-7)
Supplement: Supplementary file 2 — Supplementary Material 2. [file 12909_2025_7721_MOESM2_ESM.docx]

**Appendix A**

**Questions for Knowledge Assessment (IRAT & TRAT)**

| No. | Question: |
| --- | --- |
|  | Which of the followings are the signs of aspiration / signs suggestive of respiratory compromise of the baby in the video?   1. Sweating 2. Loud sucking sound 3. Increased stridor & work of breathing 4. Colour change/ cyanosis 5. Choking 6. (i), (iii) & (v) 7. (ii), (iii) & (v) 8. (i), (ii), (iii), (v) 9. All of the above |
|  | How many times of choking/coughing have you observed throughout the whole feed?   1. 0 time 2. 1 time 3. 2 times 4. 4 times |
|  | According to Palmer (1993), how do you describe the sucking pattern (i.e. number of sucks per sucking bursts) throughout the whole feed?   1. Mature sucking pattern 2. Dysfunctional sucking pattern, change from mature sucking pattern to immature sucking pattern 3. Disorganized sucking pattern 4. Immature sucking pattern |
|  | What is the sucking rate throughout the whole feed?   1. 1-2 sucks/sec 2. 1-4 sucks/sec 3. 3-5 sucks/ sec 4. >5 sucks/sec |
|  | Which of the followings are the possible interpretation of the baby’s action at late feed (09:45-10:00) of pushing the bottle away and arching back while showing desire to suck?   1. The baby was in distress and showed signs that he needed a rest 2. The baby wanted to continue feeding but his hands were so ‘naughty’ that he pushed the bottle away 3. The baby was hungry 4. The baby had primitive sucking reflex 5. (i), (iii), (iv) 6. (ii) & (iii) 7. (ii) & (iv) 8. (i) & (iv) |
|  | Had the baby burped after feeding?   1. Yes 2. No 3. Don’t know |

**Appendix B**

**Paediatric Feeding Simulation Clinic Skills Confidence Questionnaire**

Please rate your level of confidence in performing the following clinical skills related to pediatric dysphagia and infant feeding using the following scale:

1 Not confident at all

2 Slightly confident

3 Somewhat confident

4 Fairly confident

5 Very confident

|  | Not confident -------------------Very confident | | | | |
| --- | --- | --- | --- | --- | --- |
|  | 1 | 2 | 3 | 4 | 5 |
| Assessment | | | | | |
| I am confident in assessing and documenting an infant’s oral-motor skills and feeding behaviors. |  |  |  |  |  |
| I can identify signs and symptoms of pediatric dysphagia during a clinical assessment. |  |  |  |  |  |
| I feel confident in conducting a pediatric dysphagia evaluation. |  |  |  |  |  |
| I am able to identify red flags or indicators for further assessment in pediatric dysphagia. |  |  |  |  |  |
| Anaylsis and interpretation | | | | | |
| I am confident in analyzing and interpreting clinical data from an infant bottle-feeding assessment. |  |  |  |  |  |
| I can identify risk factors and potential causes of pediatric dysphagia based on assessment findings. |  |  |  |  |  |
| I feel confident in determining appropriate recommendations for feeding modifications or interventions based on assessment results. |  |  |  |  |  |
| I am able to communicate assessment findings and recommendations effectively to caregivers and healthcare team members. |  |  |  |  |  |
| Planning evidence-based practices for Pediatric Dysphagia | | | | | |
| I am confident in developing individualized feeding plans for infants with dysphagia. |  |  |  |  |  |
| I can select appropriate feeding strategies or therapeutic interventions for infant dysphagia management. |  |  |  |  |  |
| I feel confident in collaborating with caregivers to implement dysphagia management strategies in an infant population. |  |  |  |  |  |
| I am able to document feeding plans and interventions for pediatric dysphagia patients. |  |  |  |  |  |
| Implementation of Pediatric Dysphagia Practice | | | | | |
| I am confident in implementing feeding strategies or therapeutic exercises during infant dysphagia intervention sessions. |  |  |  |  |  |
| I can adjust intervention strategies based on real-time observations and patient responses. |  |  |  |  |  |
| I feel confident in ensuring safety and proper positioning during feeding sessions with pediatric dysphagia patients. |  |  |  |  |  |
| I am able to monitor progress and make appropriate modifications to intervention plans as needed. |  |  |  |  |  |
| Managing Pediatric Dysphagia Services | | | | | |
| I am confident in coordinating interdisciplinary care for pediatric dysphagia patients. |  |  |  |  |  |
| I can advocate for appropriate resources and support services for pediatric dysphagia management. |  |  |  |  |  |
| I feel confident in educating caregivers and healthcare providers about pediatric dysphagia prevention and management. |  |  |  |  |  |
| I am able to participate in quality improvement initiatives to enhance pediatric dysphagia services. |  |  |  |  |  |
